# Factors associated with treatment outcomes of patients with drug-resistant tuberculosis in China: A retrospective study using competing risk model

**DOI:** 10.3389/fpubh.2022.906798

**Published:** 2022-09-07

**Authors:** Zhiwei Li, Keng Lai, Tiegang Li, Zhuochen Lin, Zichao Liang, Yuhua Du, Jinxin Zhang

**Affiliations:** ^1^Department of Medical Statistics and Epidemiology, School of Public Health, Sun Yat-sen University, Guangzhou, China; ^2^Department of Tuberculosis Control and Prevention, Guangzhou Chest Hospital, Guangzhou, China; ^3^Department of Administration of Disease Prevention and Control, Guangzhou Health Committee, Guangzhou, China; ^4^Department of Medical Records, The First Affiliated Hospital, Sun Yat-sen University, Guangzhou, China

**Keywords:** factor, drug-resistant tuberculosis, treatment outcomes, LTFU, competing risk

## Abstract

**Objectives:**

Drug-resistant tuberculosis remains a serious public health problem worldwide, particularly in developing countries, including China. This study determined treatment outcomes among a cohort in Guangzhou, China, and identified factors associated with them.

**Methods:**

We initiated a retrospective study using drug-resistant TB data in Guangzhou from 2016 to 2020, managed by Guangzhou Chest Hospital. A competing risk model was used to identify the factors associated with treatment failure and death, as well as loss to follow-up (LTFU).

**Results:**

A total of 809 patients were included in the study, of which 281 were under treatment. Of the remaining 528 who had clear treatment outcomes, the number and proportion of treatment success, treatment failure, death, and LTFU were 314 (59.5%), 14 (2.7%), 32 (6.0%), and 168 (31.8%), respectively. Being older and having cavities involving the upper lungs were risk factors for treatment failure and death, while non-Guangzhou household registration and interprovincial mobility were risk factors associated with LTFU.

**Conclusion:**

Treatment failure and death were significantly associated with cavitation in the lungs, and LTFU was significantly associated with household registration and geographical mobility. Early identification of factors associated with different treatment outcomes is extremely important for policymakers, health experts, and researchers to implement appropriate strategies and measures to treat and manage the TB-infected population in China.

## Introduction

Tuberculosis (TB) is a chronic infectious disease caused by *Mycobacterium tuberculosis* (MTB), which mainly leads to lung infections but can also infect other organs. Drug-resistant TB is the result of the development of drug resistance by MTB and continues to be a public health threat (1). In 2019, nearly half a million people worldwide developed rifampicin-resistant TB (RR-TB), 78% of whom were multidrug-resistant TB (MDR-TB). The mortality and treatment failure rate of MDR-TB in HIV-negative patients were up to 21.1% and 15.0% (2), respectively. China also suffers from a relatively high burden of drug-resistant TB, as China accounted for 14% of the world's drug-resistant TB patients (1). In 2019, the number of newly diagnosed TB patients in China was 833,000, of which 7.1% were suffering from MDR/RRTB, and another 23.0% of the re-treated patients were also suffering from MDR/RRTB (1). Moreover, the burden of extensively drug-resistant tuberculosis (XDR-TB) was so heavy that it increased with an average annual percent change (AAPC) of 12.5% in prevalence, and the disease burden of TB increased with age and peaked among those aged over 70 (3).

The directly observed treatment and short-course chemotherapy (DOTS) strategy, the main treatment strategy for TB and drug-resistant TB, has been gradually rolled out globally since 1997. It was reported that the reproduction number of TB in mainland China dropped from 1.7885 to 1.0741 after DOT realized its full coverage in mainland China (4). Although good results have been achieved, they are far from enough. Studies have shown that since drug-resistant TB takes a long time to treat compared to those who are not, it is associated with more adverse reactions and more expenses (5–7), resulting in worse adherence. Therefore, the prognosis of drug-resistant patients was worse than that of non-drug-resistant patients. The incidence of loss to follow-up (LTFU) was higher, too (8).

Researchers showed some connections between treatment outcomes of drug-resistant TB and some factors. For example, Bisson et al. reported whether being infected with HIV was associated with death among multidrug-resistant (MDR) patients (9). Viboon Boonsarngsuk et al. (10) reported that the extent of radiographic disease was associated with isoniazid-, rifampicin-, and multidrug-resistant TB. Verdecchia et al. (11) found that psychosocial support and integrated care models improved the treatment success rate and reduced LTFU in populations with high proportions of MDR-TB and HIV co-infection. In most studies, the logistic regression model and the Cox proportional hazard model were used, of which the former results in large biases because survival time is not taken into consideration and the latter cannot deal with competing risks that were common in studies focused on multiple prognoses. TB is a disease with multiple treatment outcomes, such as treatment success, failure, death, and LTFU. Besides, we had an assumption that treatment failure, death, and LTFU were no longer independent of each other. In other words, competing risk occurs. Therefore, we aim to use the sub-distribution hazard model, which is a kind of competing risk model, to directly identify factors associated with its different treatment outcomes based on the cohort of drug-resistant TB patients in a representative first-tier city in China to provide targeted suggestions for the improvement of TB control strategies and directions for further research in the future.

## Patients and methods

### Study design and population

We did a retrospective study with data obtained from Guangzhou Chest Hospital, Guangdong Province, China. Guangzhou Chest Hospital is the only specialized chest hospital in Guangzhou, the capital city of Guangdong Province, with a registered resident population of 9.85 million in 2020 (12). Meanwhile, as one of the most economically developed cities in southern China, Guangzhou has a large number of floating populations. In 2020, the total number of non-registered population was 8.8 million (12). Therefore, the Guangzhou Chest Hospital provided TB diagnosis and treatment services for patients from various cities in Guangdong Province and other provinces.

**Figure 1:**
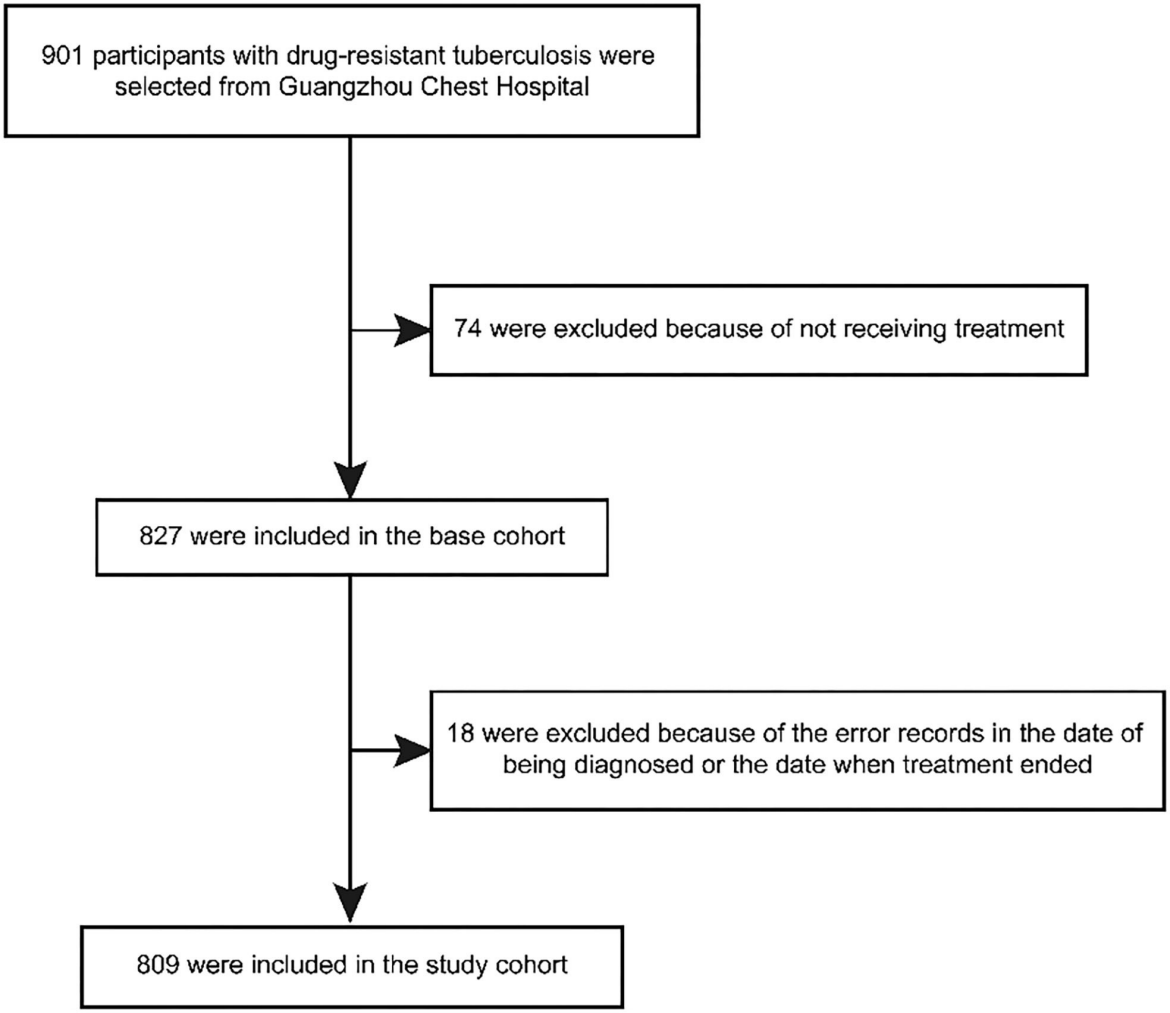
Flowchart of the study population.

We included all the patients with drug-resistant TB (defined as resistance to at least one anti-tuberculosis drug) in Guangzhou from 1 January 2016 to 31 December 2020. Based on that, patients were excluded from this study if they were (1) those who did not accept treatment; or (2) those whose dates of diagnosis were later than the dates ending their treatment due to a registration error (Figure 1).

### Diagnosis, treatment, and management

Suspected patients with TB would take bacteriological tests (sputum smear or culture) and be diagnosed according to the diagnostic criteria for TB (WS 288-2017) issued by the National Health Commission of China (13). Based on that, patients with positive cultures needed to accept drug sensitivity tests (DST). The DST was mainly carried out in Guangzhou Chest Hospital under the direction of WS 288-2017, and the results could be found in the examination system of the hospital (13).

Bacteriologically confirmed drug-resistant patients with TB would be hospitalized at Guangzhou Chest Hospital for at least 2 weeks while receiving DOTS by trained medical personnel. After that, they received outpatient treatment. All patients are treated individually, based on standardized therapies developed by WHO. During the whole process of anti-TB treatment, each patient was assigned to a designated hospital for management. Patients were managed by a trained family member or supervisor from the local community and returned to the hospital once a month for medication refills as well as adherence and progress evaluation. At the same time, staff also provide health education to patients to keep them in good adherence.

### Data source and variables

Patient information, including socio-demographic characteristics such as age, gender, occupation, household, and mobility, and clinical characteristics, such as treatment outcomes, previous TB treatment history, duration of treatment, commodities, image data, and DST results, were obtained from the internet-based TB Management Information System in the Tuberculosis Control Institute of Guangzhou and from medical records, image system as well as the testing system. In China, household registration records people's names, addresses, and other basic information for providing fast services for public life, group production, social services, and government administration. Information on household registration and mobility could be obtained directly from patients' medical records. In our study, the resistance pattern was categorized as mono/poly drug-resistant, MDR, and XDR. Mono drug-resistant is defined as resistance to only one of the first-line drugs (i.e., isoniazid, rifampicin, pyrazinamide, ethambutol, or streptomycin); poly drug-resistant is defined as resistance to two or more of the first-line drugs, but not both isoniazid and rifampicin; MDR is defined as resistance to two or more of the first-line drugs and at least both isoniazid and rifampicin; and XDR is defined as MDR plus resistance to a fluoroquinolone and an injectable agent. TB treatment history was defined as patients who have ever received anti-TB treatment, no matter what and how many drugs they have taken. The cavity was categorized as none, with cavities but not involving the upper lungs, and with cavities involving the upper lungs (bounded by the lower margin of the second costal cartilage) according to references and clinicians' experience (14).

### Treatment outcomes

Study definitions were completely consistent with WHO guidelines, as follows: treatment outcomes were classified as treatment failure, cured, treatment completed, LTFU, death, and under treatment. Treatment failure is defined as a patient with TB whose treatment was terminated or need for permanent regimen change of at least two anti-TB drugs because of the lack of conversion by the end of the intensive phase, or bacteriological reversion in the continuation phase after conversion to negative, or evidence of additional acquired resistance to fluoroquinolones or second-line injectable drugs, or adverse drug reactions (ADRs); cured is defined as a TB patient whose treatment was completed as recommended by the national policy without evidence of failure and three or more consecutive cultures taken at least 30 days apart are negative after the intensive phase; treatment completed is defined as a patient with TB whose treatment was completed as recommended by the national policy but no record of meeting the definition for cured; death is defined as a patient with TB who dies for any reason during the course of treatment; LTFU is defined as a patient with TB whose treatment was interrupted for 2 consecutive months or more; under treatment, which is equivalent to not evaluated, indicated that none of the foregoing outcomes had occurred at the end of observation (15).

The dependent variables in this study, namely, treatment outcomes, were divided into two groups: (1) treatment success if patients were cured or completed the treatment and (2) unfavorable outcomes if the patients were dead, treatment failure, or LTFU (whichever occurred first). Furthermore, unfavorable treatment outcomes were divided into two groups recursively: (2a) LTFU if the patients were “lost” during the treatment; and (2b) poor treatment outcomes if the patients died or had an outcome of treatment failure.

### Data analysis

Continuous variables were described using the mean and standard deviation if they were normally distributed, otherwise the median and interquartile range would be utilized. The classification variables are described using frequency and percentage. The cumulative incidence function (CIF) was used to estimate the cumulative incidence rate of various events (i.e., poor treatment outcomes or LTFU). The Fine and Gray models were used to determine the influential factors associated with treatment outcomes.

The time to treatment outcome is the period from when a patient is diagnosed as drug-resistant to when treatment outcome occurs. In our analysis, both successful treatment outcomes (i.e., cured and treatment completed) and under treatment (i.e., no treatment outcomes mentioned above occurred by 31 December 2020) were defined as censored. In our study, we focused on two different events based on the competing risk framework. Event one was the occurrence of poor treatment outcomes (death or treatment failure, whichever occurred first), and event two was the occurrence of LTFU. In the analysis of poor treatment outcomes, we considered LTFU as a competing risk (i.e., events that occur instead of the event of interest). Similarly, in the analysis of LTFU, death was considered a competing risk. Due to the existence of competing risks, we used the Fine and Gray subdistribution model based on CIF, which can directly estimate the effects of the factors associated with the event of interest in the presence of competing risks.

For all the complete cases, univariate analysis was performed for the two events individually. As a result, factors whose *P*-value < 0.10 and age (we include age as a continuous variable rather than divide it into several age groups) were included in the multivariate analysis. We also included hypertension, diabetes, and resistance pattern as confounders that needed to adjust no matter if they are statistically significant or not. Factors with *P* < 0.05 in multivariate analysis were considered statistically significant. Then, a sensitivity analysis using the 500 data generated by multiple imputations was run based on the assumption that missing values were missing at random. The covariates used to impute missing variables were all the complete variables except the dependent variables. We adopted the multivariate model established by complete case analysis to analyze these datasets, and we got different estimates of the same covariates. Finally, according to Rubin's Rule, we pooled these results.

Data recording was done with Microsoft Excel 2016, and analyses were fulfilled using R 4.0.2 with the package “cmprsk.”

## Results

### Treatment outcomes

A total of 809 patients with drug-resistant TB were included in the study from 1 January 2016 to 31 December 2020. Table 1 shows the distribution of treatment outcomes for all the patients. Among the 809 patients, 281 were under treatment, and of the remaining 528 who had clear treatment outcomes, the number and proportion of treatment success, treatment failure, death, and LTFU were 314 (59.5%), 14 (2.7%), 32 (6.0%), and 168 (31.8%), respectively.

**Table 1:** Distribution of treatment outcomes in 809 patients with drug-resistant tuberculosis in Guangzhou, China, from 2016 to 2020.

**Outcome**	**Frequency (*n*)**	**Percentage (%)**
Treatment success	314	38.8
Treatment failure	14	1.7
Death	32	4.0
LTFU	168	20.8
Under treatment	281	34.7
Total	809	100.0

### Demographic characteristics of patients

As shown in Table 2, among 809 patients, 584 were men, accounting for 72.2%. Farmers accounted for 21.9% (7/32) of the dead patient group, which was higher than other groups. LTFU was low in the proportion of the registered population in Guangzhou and high in interprovincial mobility, accounting for 26.2% (44/168) and 9.5% (16/168), respectively. The use of alcohol and tobacco was comparable among all the treatment outcomes. The median age at diagnosis of all the patients was 44 years old (*IQR*: 31–57) and 62 years old (*IQR*: 56–71.5) in the dead patient group.

**Table 2:** Summary of patient characteristics and their treatment outcomes.

**Variable**	**Treatment success**	**Treatment failure**	**Death**	**LTFU**	**Under treatment**	**Total**
	***N* (%)**	***N* (%)**	***N* (%)**	***N* (%)**	***N* (%)**	***N***
**Gender**						
Female	98 (31.2)	2 (14.3)	5 (15.6)	42 (25.0)	78 (27.8)	225 (27.8)
Male	216 (68.8)	12 (85.7)	27 (84.4)	126 (75.0)	203 (72.2)	584 (72.2)
**Occupation**						
Other	283 (90.1)	13 (92.9)	25 (78.1)	151 (89.9)	257 (91.5)	729 (90.1)
Farmer	31 (9.9)	1 (7.1)	7 (21.9)	17 (10.1)	24 (8.5)	80 (9.9)
**Household registration**						
Guangzhou	146 (46.5)	5 (35.7)	28 (87.5)	44 (26.2)	161 (57.3)	384 (47.5)
Non-Guangzhou	168 (53.5)	9 (64.3)	4 (12.5)	124 (73.8)	120 (42.7)	425 (52.5)
**Previous TB treatment history**						
No	71 (22.6)	3 (21.4)	10 (31.2)	62 (36.9)	161 (57.3)	307 (37.9)
Yes	243 (77.4)	11 (78.6)	22 (68.8)	106 (63.1)	120 (42.7)	502 (62.1)
**Diabetes**						
No	270 (86.0)	9 (64.3)	21 (65.6)	123 (73.2)	227 (80.8)	650 (80.3)
Yes	38 (12.1)	4 (28.6)	10 (31.3)	31 (18.5)	52 (18.5)	135 (16.7)
Unknown	6 (1.9)	1 (7.1)	1 (3.1)	14 (8.3)	2 (0.7)	24 (3.0)
**Smoking**						
No	237 (75.5)	10 (71.4)	22 (68.8)	118 (70.3)	208 (74.0)	595 (73.5)
Yes	71 (22.6)	3 (21.4)	9 (28.1)	35 (20.8)	70 (24.9)	188 (23.2)
Unknown	6 (1.9)	1 (7.2)	1 (3.1)	15 (8.9)	3 (1.1)	26 (3.3)
**Drinking**						
No	285 (90.8)	12 (85.8)	27 (84.4)	141 (83.9)	247 (87.9)	712 (88.0)
Yes	23 (7.3)	1 (7.1)	4 (12.5)	12 (7.2)	31 (11.0)	71 (8.8)
Unknown	6 (1.9)	1 (7.1)	1 (3.1)	15 (8.9)	3 (1.1)	26 (3.2)
**Viral hepatitis**						
No	288 (91.7)	12 (85.8)	30 (93.8)	148 (88.1)	263 (93.6)	741 (91.6)
Yes	20 (6.4)	1 (7.1)	1 (3.1)	5 (3.0)	15 (5.3)	42 (5.2)
Unknown	6 (1.9)	1 (7.1)	1 (3.1)	15 (8.9)	3 (1.1)	26 (3.2)
**Hypertension**						
No	303 (96.5)	13 (92.9)	28 (87.5)	151 (89.9)	262 (93.2)	757 (93.6)
Yes	5 (1.6)	0 (0)	3 (9.4)	2 (1.2)	16 (5.7)	26 (3.2)
Unknown	6 (1.9)	1 (7.1)	1 (3.1)	15 (8.9)	3 (1.1)	26 (3.2)
**Mobility**						
Intra-urban	241 (76.8)	9 (64.3)	31 (96.9)	123 (73.2)	277 (98.6)	681 (84.2)
Inter-city	67 (21.3)	4 (28.6)	1 (3.1)	29 (17.3)	4 (1.4)	105 (13.0)
Interprovincial	6 (1.9)	1 (7.1)	0 (0)	16 (9.5)	0 (0)	23 (2.8)
**Resistance pattern**						
Mono/Poly drug-resistant	45 (14.4)	1 (7.2)	5 (15.5)	42 (25.0)	49 (17.4)	142 (17.6)
MDR	250 (79.6)	10 (71.4)	22 (68.8)	114 (67.8)	188 (66.9)	584 (72.2)
XDR	17 (5.4)	3 (21.4)	2 (6.3)	8 (4.8)	10 (3.6)	40 (5.0)
Unknown	2 (0.6)	0 (0)	3 (9.4)	4 (2.4)	34 (12.1)	43 (5.2)
**Cavity**						
None	147 (46.8)	4 (28.6)	5 (15.6)	69 (41.1)	119 (42.3)	344 (42.5)
With the cavities not involving the upper lungs	17 (5.4)	0 (0)	1 (3.1)	8 (4.8)	20 (7.1)	46 (5.7)
With the cavities involving the upper lungs	140 (44.6)	9 (64.3)	23 (71.9)	78 (46.4)	135 (48.1)	385 (47.6)
Unknown	10 (3.2)	1 (7.1)	3 (9.4)	13 (7.7)	7 (2.5)	34 (4.2)
Age	40 (30, 56)	40 (29.5, 54)	62 (56, 71.5)	47 (36, 58)	43 (29, 56)	44 (31, 57)

MDR, multidrug-resistant; XDR, extensively drug-resistant.

### Clinical characteristics of patients

In the group of treatment failure and death, the proportion of those with cavities involving the upper lungs was 64.3% (9/14) and 71.9 (23/31) and their diabetes prevalence was a little higher than others, accounting for 28.6% (4/14) and 31.3 (10/32), respectively. Besides, it seems that there were not many differences in the prevalence of viral hepatitis and hypertension as well as the proportion of those receiving TB treatment previously among treatment success, treatment failure, death, and LTFU. Of all the 809 patients, the proportion of MDR is the highest, accounting for 72.2% (584/809), followed by mono/poly drug-resistant and XDR, accounting for 17.6% (142/809) and 5.0% (40/809), respectively.

There were missing values in diabetes, smoking, drinking, viral hepatitis, hypertension, resistance pattern, and cavities. The one with the highest missing rate was the resistance pattern, at 5.2% (43/809).

### Survival analysis

**Figure 2:**
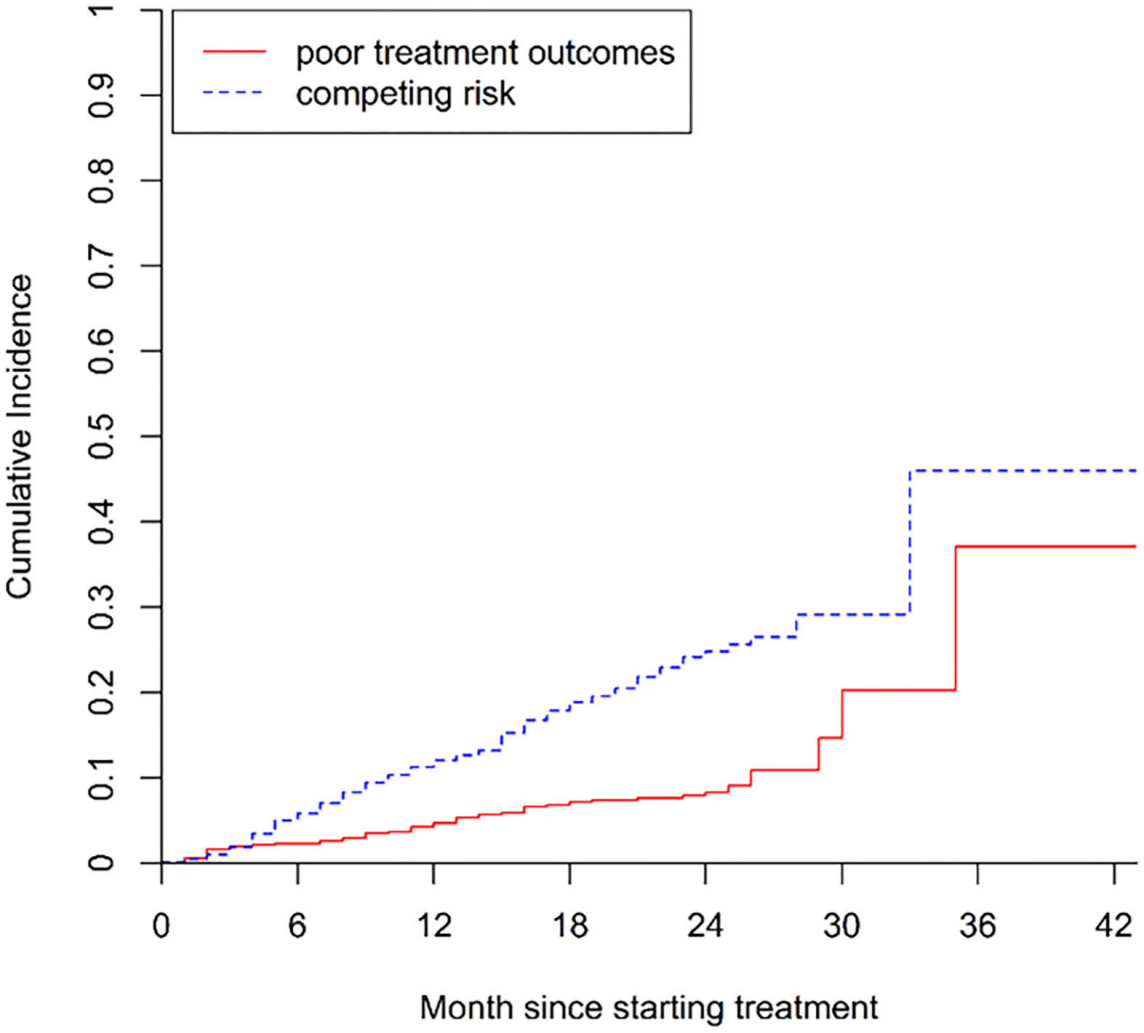
Cumulative incidence of poor treatment outcomes (i.e., treatment failure and death) in patients with drug-resistant tuberculosis in Guangzhou, China, from 2016 to 2020.

**Figure 3:**
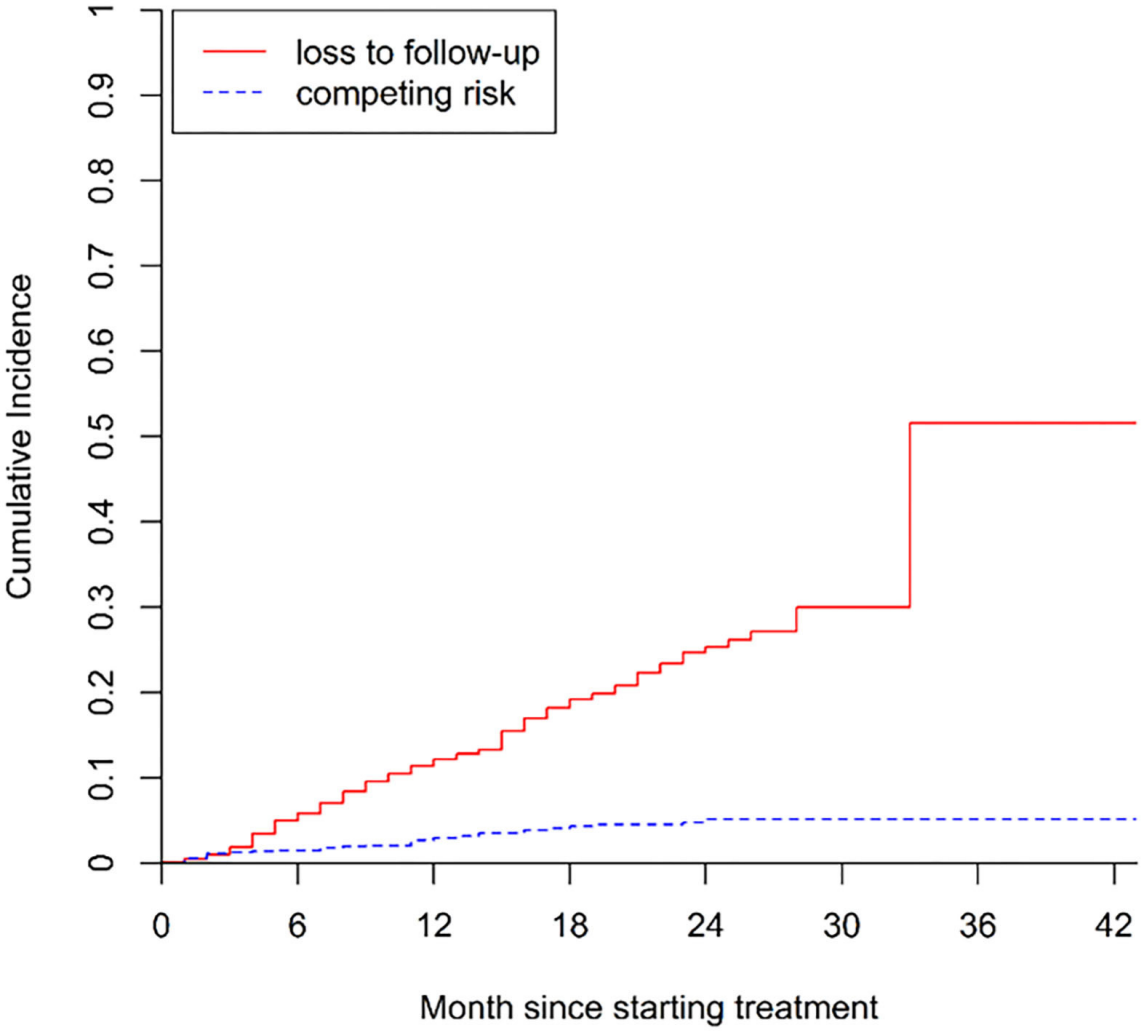
Cumulative incidence of LTFU (i.e., loss to follow-up) in patients with drug-resistant tuberculosis in Guangzhou, China, from 2016 to 2020.

The cumulative incidence of poor treatment outcomes (event one) among all patients at months 6, 12, and 24 were 2.3, 4.7, and 8.3%, respectively (Figure 2). At months 6, 12, and 24, the cumulative incidence of LTFU were 5.9, 12.2, and 25.4%, respectively (Figure 3). The median follow-up time of patients who discontinued treatment was 302 days (IQR: 146.8–484), and 74.7% (115/154) of LTFU dropped out after their intensive phase (defined as 6 months after starting treatment).

### Factors associated with poor treatment outcomes and LTFU

We first conducted a multivariate analysis of the two events based on 722 complete cases. When focusing on event one, being older and having a cavity involving the upper lungs were risk factors (HR = 1.04, 95% CI: 1.02–1.05; HR = 1.90, 95% CI: 1.00–3.61) (Table 3). In the analysis of event two, non-Guangzhou household registration (HR = 2.81, 95% CI: 1.78–4.42) and interprovincial mobility (HR = 2.73, 95% CI: 1.44–5.15). Although with a *P*-value of 0.071, the actual lower limit of 95% CI of age was 0.999, making it very close to statistically significant (Table 4).

**Table 3:** Factors associated with poor treatment outcomes (i.e., death or failure) in patients with drug-resistant tuberculosis in Guangzhou, China, from 2016 to 2020 in complete case analysis and sensitivity analysis after multiple imputations.

**Variables**	**Complete cases analysis**		**Sensitivity analysis**	
	**HR (95% CI)**	***P-value***	**HR (95% CI)**	***P-value***
Age	1.04 (1.02–1.05)	<0.001^*^	1.04 (1.02–1.06)	<0.001^*^
**Household registration**				
Guangzhou	Reference		Reference	
Non-Guangzhou	0.90 (0.51–1.59)	0.720	0.86 (0.51–1.46)	0.582
**Diabetes**				
No	Reference		Reference	
Yes	1.65 (0.87–3.12)	0.120	1.52 (0.83–2.79)	0.172
**Hypertension**				
No	Reference		Reference	
Yes	1.00 (0.24–4.18)	1.000	0.95 (0.29–3.13)	0.932
**Cavity**				
None	Reference		Reference	
With the cavities not involving the upper lungs	0.52 (0.07–4.04)	0.530	0.54 (0.07–4.19)	0.554
With the cavities involving the upper lungs	1.90 (1.00–3.61)	0.049^*^	1.86 (1.02–3.41)	0.044^*^
**Resistance pattern**				
Mono/Poly drug-resistant	Reference		Reference	
MDR	0.69 (0.34–1.38)	0.290	0.69 (0.37–1.28)	0.239
XDR	1.13 (0.37–3.41)	0.830	1.30 (0.50–3.40)	0.588

* Significant at α = 0.05.

HR, hazard ratio; CI, confidence interval; MDR, multidrug-resistant; XDR, extensively drug-resistant.

**Table 4:** Factors associated with LTFU (i.e., loss to follow-up) in patients with drug-resistant tuberculosis in Guangzhou, China, from 2016 to 2020 in complete case analysis and sensitivity analysis after multiple imputations.

**Variables**	**Complete cases analysis**		**Sensitivity analysis**	
	**HR (95% CI)**	***P-value***	**HR (95% CI)**	***P-value***
Age	1.01 (1.00–1.02)^⊥^	0.071	1.01 (1.00–1.02)	<0.001*
**Household registration**				
Guangzhou	Reference		Reference	
Non-Guangzhou	2.81 (1.78–4.42)	<0.001*	2.93 (1.94–4.42)	<0.001*
**Diabetes**				
No	Reference		Reference	
Yes	1.51 (0.94–2.41)	0.086	1.93 (0.88–2.20)	0.158
**Hypertension**				
No	Reference		Reference	
Yes	0.29 (0.04–2.18)	0.230	0.47 (0.12–1.83)	0.274
**Mobility**				
Intra-urban	Reference		Reference	
Inter-city	0.91 (0.57–1.46)	0.700	0.87 (0.56–1.37)	0.561
Interprovincial	2.73 (1.44–5.15)	0.002*	2.71 (1.55–4.72)	<0.001*
**Resistance pattern**				
Mono/Poly drug-resistant	Reference		Reference	
MDR	0.63 (0.40–1.01)	0.055	0.60 (0.40–0.90)	0.013*
XDR	0.82 (0.36–1.86)	0.630	0.66 (0.29–1.50)	0.324

^⊥^The actual lower limit of 95% CI is 0.999.

^*^Significant at α = 0.05.

HR, hazard ratio; CI, confidence interval; MDR, multidrug-resistant; XDR, extensively drug-resistant.

Sensitivity analyses showed that after multiple imputations, the results for events one and two were almost identical to those of the complete data analysis, except that age (HR = 1.01, 95% CI: 1.00–1.02) was a risk factor and MDR (HR = 0.60, 95% CI: 0.40–0.90) was a protective factor for LTFU after multiple imputations.

## Discussion

Unlike previous studies focusing on a comprehensive unfavorable outcome, which is the composite event of treatment failure, death, and LTFU, this study focused on the outcome of treatment failure and death, as well as the LTFU separately by using a competing risk model. In our study, the proportion of LTFU was 31.8%, slightly higher than that of 29% in the TB report 2020 of WHO and 27% in a study of Hunan province, China, in 2017 (1, 16). The proportion of treatment success in our study was 59.5%, which was slightly higher than that of 56% globally in 2016 (1) and 59% reported in a larger study in Chongqing, China (17). These facts suggest that the treatment and management of drug-resistant TB in Guangzhou, China, might perform above the average. However, there is room for improvement because this relatively low rate of treatment success and high rate of LTFU are still threats to TB control, as drug-resistant patients may develop higher levels of resistance or transmit drug-resistant TB to others.

Through our study, we had some findings that may have implications for drug-resistant TB management. To begin with, older age was a risk factor in the analysis for the poor treatment outcomes. This result was consistent with some other studies (18–20). It was reported that the cavity is an independent factor associated with poor treatment outcomes (21). The cavity is meaningful for the treatment and transmission of drug-resistant tuberculosis. Urbanowski et al. (22) pointed out that one of the mechanisms of treatment failure and death in tuberculosis is that the tubercle bacillus is highly loaded in the cavities and easy to proliferate. Kempker et al. (23) also pointed out that *Mycobacterium tuberculosis* in cavities is more resistant to drugs. In our study, cavities involving the upper lungs were found to be a risk factor. The cavity is a risk factor for inducing resistance and leading to treatment failure, yet considering its location at the same time is the highlight of this study. Bowness et al. (24) demonstrate that when bacteria are located further away from blood vessels, less favorable outcomes including treatment failure are more likely to happen, which is a hint for why cavities located in the upper lungs might lead to failure more easily because the blood supply to the upper lungs is poor in general. We believe that such a clear classification will be a good reminder for clinicians' judgment and convenience for professionals in the field of TB prevention and control.

The consideration of household registration and mobility is an interesting point in our study. In the analysis of LTFU, we found that the loss of follow-up was more related to demographic characteristics, especially geographical information – non-Guangzhou household registration and interprovincial mobility. This may be an unexpected finding, as most researchers may tend to assume that LTFU may have opted out of treatment due to adverse drug reactions or unexpected clinical events. Loss of follow-up is an important phenomenon in the treatment duration of drug-resistant patients with TB. Due to the long treatment duration of drug-resistant tuberculosis and the relatively poor prognosis, the occurrence of loss of follow-up is common. When it occurs, it means that the management unit is unable to know the progress of patients. A high incidence of loss of follow-up was not only associated with the potential risk of transmission but also might lead to the waste of medical resources. In July 2014, after the MDT-TB treatment project of the Global Fund was completed, Guangzhou formulated the prevention and treatment strategy for MDR-TB. Because of the relative limitation of medical resources, drug-resistant patients who were Guangzhou residents can get preferential treatment, while others cannot accept this preferential treatment (25). Therefore, household registration and mobility are substantially linked to economic factors in this cohort. Silent transfer, which means that patients would voluntarily choose a medical institution that is convenient to them for treatment (26), especially in a long-term follow-up, might be the reason for the drop-out phenomenon in our study. This convenience might be due to lower medical expenses and a less crowded environment. From this point of view, it is reasonable to speculate that the high expense of health care and the distributive imbalance of health resources were the deeper factors that promoted the occurrence of loss to follow-up in patients with drug-resistant TB. Guangdong Province, where Guangzhou is located, has the largest economic output in China, while most of the drug-resistant TB patients who came to Guangzhou Chest Hospital from other provinces were from neighboring provinces, such as Hunan and Jiangxi provinces, which are relatively backward in economic development. It was no surprise that they would prefer to receive inefficient treatment in their hometown rather than migrate to Guangzhou for more expensive treatment, even though the treatment in Guangzhou would be much more effective. Although strengthening information communication among different regions is the most effective way to reduce the incidence of LTFU, it is also meaningful to optimize the management of funds. Guangdong Province is a province with a large floating population, where 47.3% of its permanent residents are non-registered. Therefore, it should be encouraged to strengthen the treatment allowance for non-registered patients.

Sensitivity analysis showed that most of the estimations in our study were robust, except for age and resistance pattern for analysis of LTFU. Age turned out to be statistically significant in sensitivity analysis, which was not the case in the complete case analysis. It was likely that the complete case analysis underestimated the effect of age by removing observations containing missing values. This result is in line with what Patra et al. (27) reported. Studies have shown that TB patients are more likely to discontinue treatment when they feel their symptoms are alleviating or they just got better (28–30). In other words, more severe illness may make it easier for patients to comply with treatment and prevent them from dropping out. In our study, more than 70% of LTFUs dropped out after their intensive phase. It is possible that during the intensive period, patients who were MDR or XDR shell had slower remission and thus felt worse than those who were mono/poly drug-resistant, which in turn led to better compliance. Therefore, to minimize the phenomenon of loss to follow-up caused by the improper subjective judgment of patients, DOTS health education should be firmly implemented.

This study is subject to several limitations. First, this is a retrospective study, and because treating drug-resistant TB is a lengthy process, some data, such as the results of sputum smear culture, were not well collected or preserved during patient treatment. Apart from this, the information on the duration and severity of TB was unavailable. Since a large proportion of patients in our dataset had taken anti-tuberculosis treatment previously and it is difficult to trace back the past treatment records, it is hard to know the duration of TB among those patients. Besides, the severity of tuberculosis is mainly judged by a combination of chest X-ray findings, pathogenic findings, and clinical symptoms, which cannot be evaluated from a single index and thus is difficult to quantify the information on the severity of TB unavailable. We expect that future research can take these factors into account and improve the data quality of the study. Second, we did not include information about patients' treatment regimens. In the implementation of DOTS, outpatients were required to take their medications regularly and fill out medication record cards. At the same time, their compliance and health status were evaluated when they returned to the management unit regularly to pick up their medications. As a result, patients' compliance with treatment was generally good. In addition, treatment regimens would be subjected to appropriate changes in the context of good patient-physician interactions but are generally in line with the basic regimen. Therefore, the absence of treatment regimens may have little impact on our study, while an improved approach to recording patient data and completing clinical information would help to further understand the factors associated with treatment outcomes for patients with drug-resistant TB. Such knowledge would also help to develop appropriate interventions to improve the prognostic regression of patients. Finally, in China, patients with HIV co-infection are treated in specialized infectious disease hospitals rather than in chest hospitals. Patients with HIV co-infection were excluded in our study. We noticed that previous studies had shown that HIV co-infection is an important risk factor for drug-resistant TB (31–33).

## Conclusion

This study showed sub-optimal treatment success rates among patients with drug-resistant TB and high rates of LTFU in Guangzhou, Guangdong Province, China. Furthermore, this study found that age and cavity were risk factors associated with treatment failure and death, while LTFU was directly related to geographical factors such as household registration and mobility. Specific policies and measures should be designed to reduce the high rate of poor treatment outcomes and LTFU.

## Data availability statement

The data analyzed in this study is subject to the following licenses/restrictions: Data was obtained from Guangzhou Chest Hospital and are available with the permission of Guangzhou Chest Hospital on reasonable request. Requests to access these datasets should be directed to lizhw58@mail2.sysu.edu.cn.

## Ethics statement

The studies involving human participants were reviewed and approved by the Ethics Committee of School of Public Health, Sun Yat-sen University. Written informed consent from the participants' legal guardian/next of kin was not required to participate in this study in accordance with the national legislation and the institutional requirements.

## Author contributions

Conceptualization and project administration: JZ and TL. Methodology and software: ZLi and ZLin. Formal analysis: ZLi and KL. Writing—original draft: ZLi. Writing—reviewing and editing: ZLi, JZ, and TL. Data curation and investigation: KL and ZLia. Validation: ZLin. Resources and supervision: YD. Funding acquisition: JZ. All authors contributed to the article and approved the submitted version.

## Funding

This study was funded by the National Science and Technique Major Project of China (Grant Number 2018ZX10715004).

## Conflict of interest

The authors declare that the research was conducted in the absence of any commercial or financial relationships that could be construed as a potential conflict of interest.

## Publisher's note

All claims expressed in this article are solely those of the authors and do not necessarily represent those of their affiliated organizations, or those of the publisher, the editors and the reviewers. Any product that may be evaluated in this article, or claim that may be made by its manufacturer, is not guaranteed or endorsed by the publisher.
